# Denosumab combined with microwave ablation excisional scraping for giant cell tumor of the thoracic spine: a case report and literature review

**DOI:** 10.3389/fonc.2024.1402550

**Published:** 2024-09-19

**Authors:** Zukang Miao, Zhiwei Han, Kai Zheng, Ming Xu, Xue Yu, Changsheng Han, Xiuchun Yu

**Affiliations:** ^1^ Department of Orthopaedic Surgery, The 960th Hospital of the People’s Liberation Army Joint Logistics Support Force, Jinan, Shandong, China; ^2^ Department of Joint Surgery, Central Hospital Affiliated to Shandong First Medical University, Jinan, Shandong, China; ^3^ School of Anesthesia, Shandong Second Medical University, Weifang, Shandong, China

**Keywords:** denosumab, excisional scraping, giant cell tumor of bone, case report, literature review

## Abstract

Denosumab has recently become an important part of the treatment regime for spinal giant cell tumors of the bone (GCTB). Its use has significantly reduced the risk of surgery and postoperative complications in patients with spinal GCTB. However, the use of denosumab has not yet been optimized to reap the maximum benefits. Here, we have reported the case of a patient who was treated with denosumab in combination with excision and scraping for GCTB of the T10 vertebrae, which achieved good tumor control and no recurrence at the 2-year postoperative follow-up. We have also reviewed the case in the light of relevant literature as well as presented our ideas and recommendations for the optimal use of denosumab.

## 1 Introduction

Giant cell tumor of the bone (GCTB) is an aggressive tumor with a very high recurrence rate (1). The most common sites of its occurrence are the long bones of the extremities, with 1–5% involvement of the spine (including the vertebrae, pelvis, and sacrum) (2). For spinal GCTB, surgery is the sure-fire treatment approach and it includes total *en bloc* spondylectomy and intra-lesional curettage. Total *en bloc* spondylectomy (TES) is the treatment of choice for spinal GCTB; however, considering the special anatomy of the spine, its location, and the extent of the tumor, serious complications may occur (3). Relatively, intra-lesional curettage surgery is less traumatic for patients and has fewer postoperative complications, albeit the chance of tumor recurrence is 15–30% (4). Therefore, it is important to achieve complete curettage, reduce the postoperative recurrence rates, and establish an effective adjuvant treatment for GCTB in difficult-to-treat areas.

Denosumab is a human immunoglobulin (Ig) G2 monoclonal antibody that targets and inhibits RANKL activation. It functions similarly to osteoprotegerin (OPG), which inhibits osteoclast activation and differentiation by competitively binding to RANKL, which, in turn, reduces bone resorption and increases bone mass (5). The Chinese Society of Clinical Oncology (CSCO) guidelines mention the treatment of GCTB (Class 2A evidence). However, there are still some unresolved issues regarding the use of denosumab in clinical practice, including the rapid recurrence after discontinuation of the drug after long-term use and the use of the drug before curettage as it may increase the risk of local recurrence (6). Therefore, it remains elusive as to how denosumab should be used to maximize patient benefit. Here, we have reported the case of a patient who underwent denosumab treatment in combination with excision and scraping for GCTB in T10 vertebrae; the patient showed no tumor recurrence or uncomfortable symptoms at 24-month postoperative follow-up, indicating that the intervention achieved good clinical outcome.

## 2 Case report

**Figure 1:**
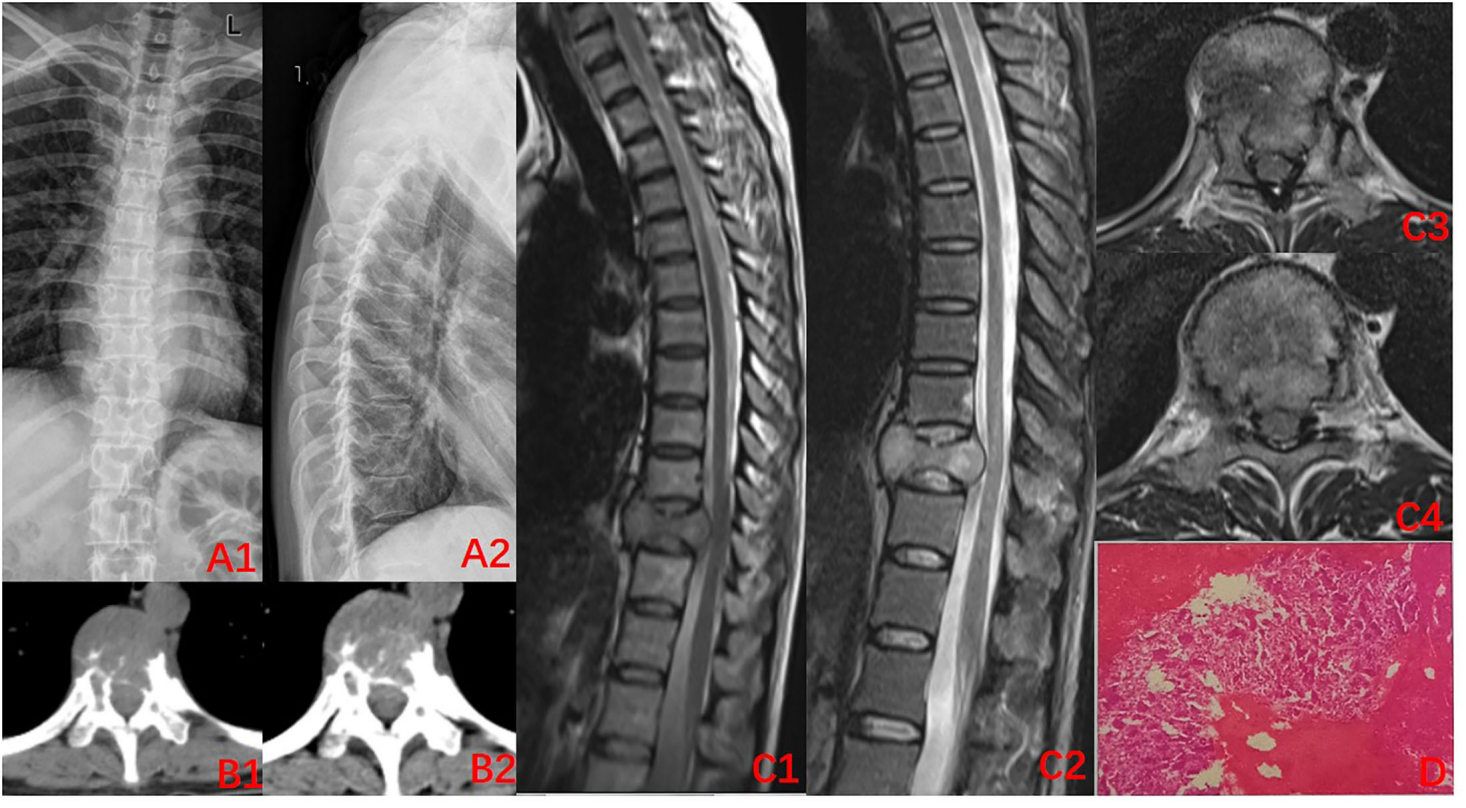
Imaging at the time of initial diagnosis (2022–01). **(A1-B2)** X-ray of the thoracic spine. CT: Decreased density of T10 vertebral body, destruction of the vertebral bone, and discontinuity of the bone cortex; **(C1–C4)** MRI of the thoracic spine: centric flattening of the T10 vertebral body, heterogeneous signal changes within the vertebral body, dural compression, and narrowing of the spinal canal; **(D)** Puncture pathology: a large number of osteoclasts can be seen with the invasion of normal bone tissue, indicative of giant cell tumor of the bone.

A 45-year-old woman was admitted to our hospital on January 21, 2022, with the complaint of “lower back pain and restricted movement for more than 2 months”. Her pre-existing physical fitness revealed no family history of genetic disorders. Specialist examination showed no evident deformity of the spine, limited flexion, extension, and rotation. Significant pressure and percussion pain in the thoracolumbar region were noted, albeit there was no radiating pain. Normal skin sensation of the trunk and lower limbs, normal muscle strength, and tone of both lower limbs were recorded. Physiological reflexes were present, while pathological reflexes were not elicited. Her blood tests showed no significant abnormalities. Imaging, X-ray, and computed tomography (CT) revealed an irregular morphology of the T10 vertebral body with abnormal bone destruction. The magnetic resonance imaging (MRI) displayed flattening of the T10 vertebral body with uneven signal changes and compression of the spinal canal (**Figures 1A1–C4**). CT-guided aspiration biopsy of the T10 vertebral tumor showed aspiration pathology, indicating GCTB (**Figure 1D**). The cumulative observations of the abovementioned clinical manifestations and examination outcomes led to the preliminary diagnosis of T10 vertebrae GCTB.

**Figure 2:**
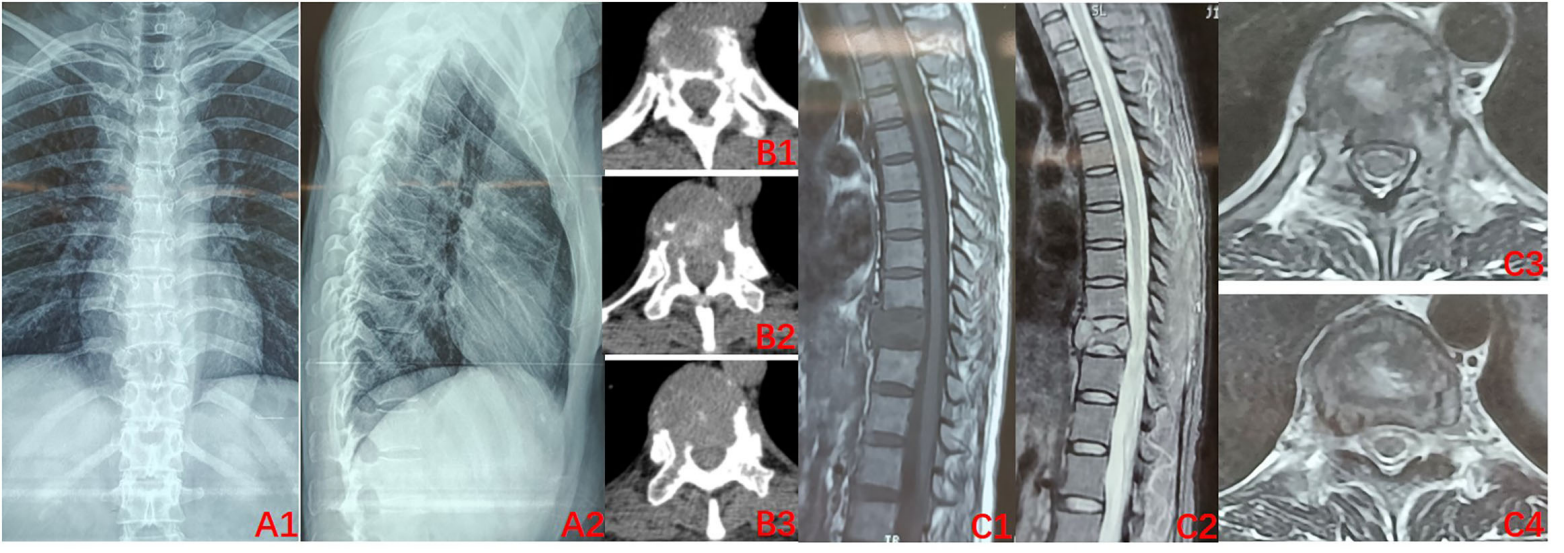
Preoperative imaging after 3 doses of denosumab (2022–03). **(A1–B3)** X-ray of the thoracic spine. CT: Bone density of the T10 vertebral body was significantly improved relative to that previously, uneven ossification was observed within the vertebral body, and bone formation was evident at the posterior margin of the vertebral body; **(C1–C4)** MRI of the thoracic spine: vertebral signal inhomogeneity was altered, tumor size is reduced, and intravertebral occupancy was improved.

**Figure 3:**
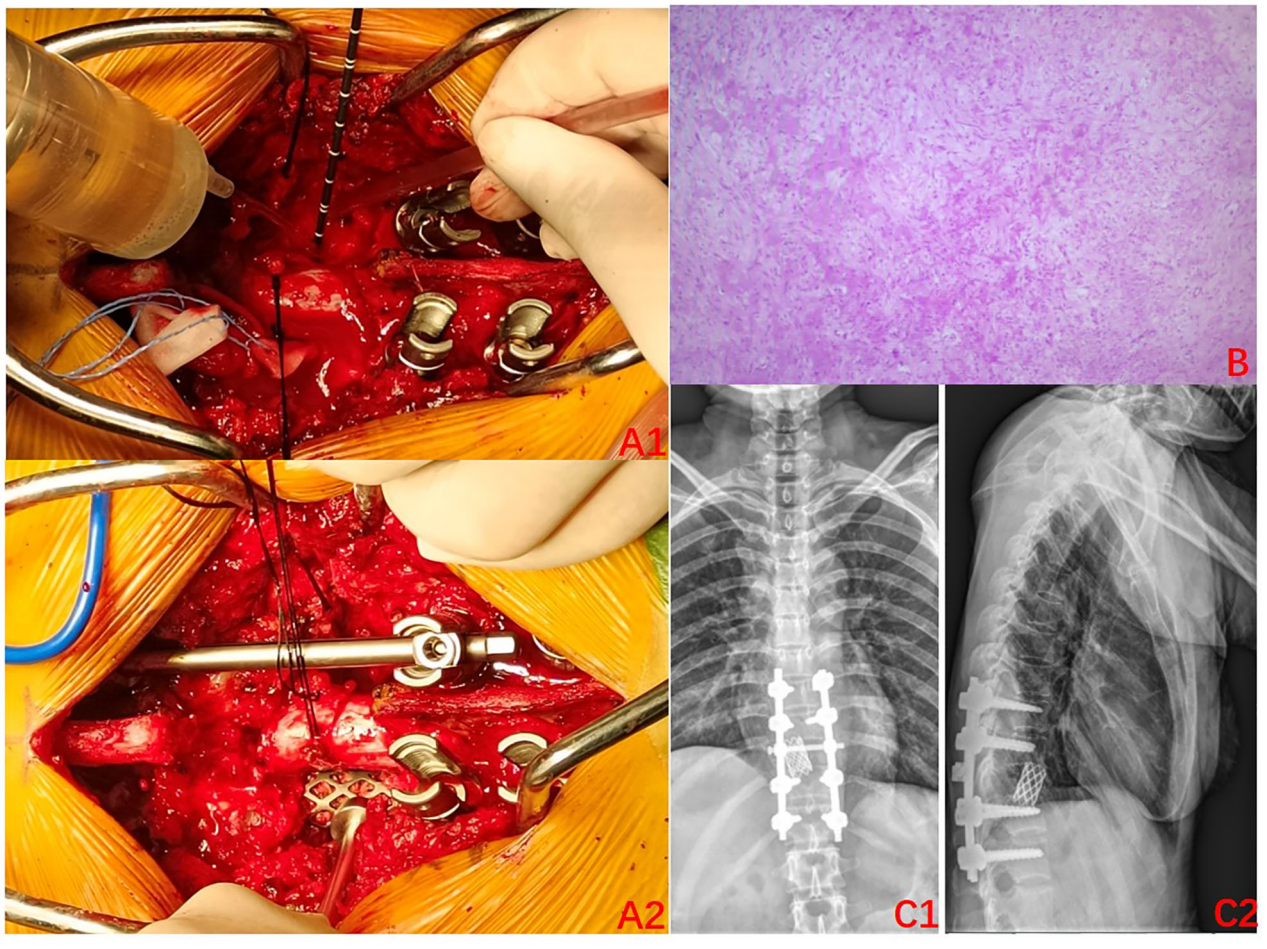
Surgery-related imaging. **(A1)** Intraoperative photos (2022–03): Microwave inactivation of vertebral tumors; **(A2)**: Implanted titanium cage;. **(B)** Postoperative pathology: focal new bone formation can be seen, with some areas of degenerative-like necrosis of the bone tissues. **(C1–C2)** Postoperative X-rays (2022–04); A satisfactory position of the internal fixation can be seen.

The condition and associated risks were explained in detail to the patient and his family, who then agreed to the treatment plan of using the drugs in combination with surgery. Three separate subcutaneous injections of 120 mg denosumab were administered on days 1, 8, and 15 along with the concomitant oral administration of calcium carbonate D3, 600 mg, qd. After 3 weeks of medication, imaging was reviewed: the T10 vertebral body tumor was observed to be significantly smaller than earlier, partially ossified (**Figures 2A1–C4**). A microwave inactivation and scraping of the T10 vertebral body tumor via the posterior approach was performed on March 8, 2022, for internal fixation with bone grafting. After routine intraoperative incision and exposure, exposure of the T8-T12 bilateral articular processes and vertebral plates, pedicle screws of an appropriate length were implanted in T8, T9, T11, and T12, and a titanium rod of an appropriate length was placed on one side for temporary fixation, followed by ultrasonic osteotome cutting of the T10 plate, resection of T9 inferior articular process and T10 superior articular process and exposure of the dura mater and nerve roots, and cutting of the T10 nerve root. A microwave needle was then inserted into the T10 vertebral body through one side of the pedicle, with the microwave needle setting power at 60 W; the microwave inactivation of the tumor was conducted thrice unilaterally at multiple points and directions, while ice-water rinsing was performed, and the thermometer needle was continuously measured to maintain a temperature <42°C to protect the spinal cord, while the contralateral side was treated similarly for a total of six times. After microwave inactivation, the vertebral body was cut and scraped from both sides to achieve complete resection, and the lower endplate of T9 and the upper endplate of T11 were processed to expose the normal oozing bone. Then implantation of allograft bone at the anterior margin of the vertebral body a titanium cage filled with allograft bone of the appropriate length was placed. After the examination of the c-arm X-ray machine, the position of the internal fixation is satisfactory, a titanium rod was implanted, the drainage was left in place, and the incision was sutured (**Figures 3A1, A2**). The postoperative pathology was consistent with GCTB (**Figure 3B**).

**Figure 4:**
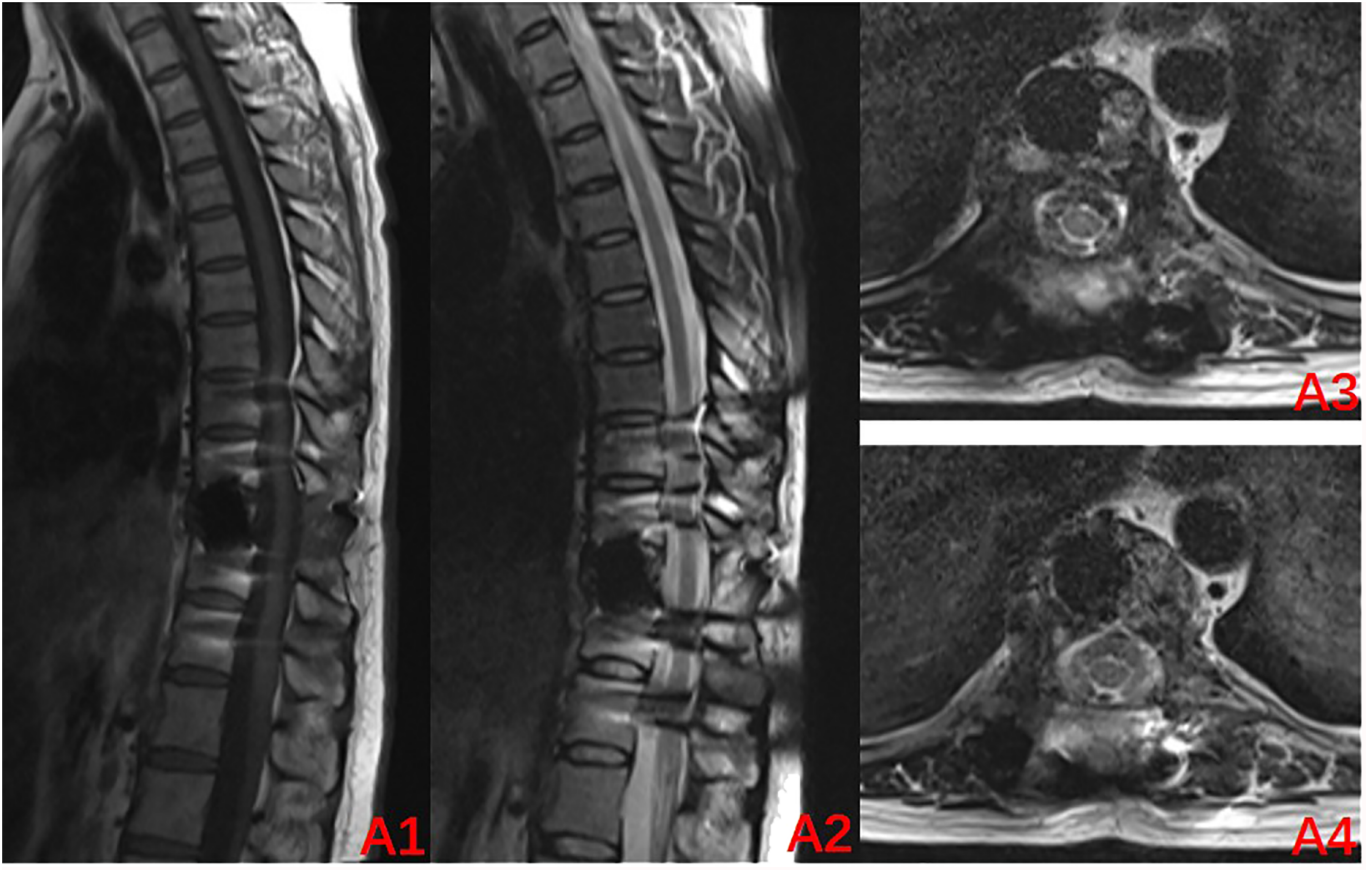
Review MRI at 24 months after the surgery (2024–03): **(A1–A4)** Internal fixation artifacts can be seen in the T10 vertebral body, with no abnormal signal in the spinal canal and a normal sagittal diameter.

The radiographs were reviewed 1 month after the surgery and displayed a satisfactory position of the internal fixation (**Figures 3C1, C2**), as such the use of denosumab was resumed. It was planned to be used every 3 months for the first 2 years after the surgery and then every 6 months in the third year. Finally, it was used once every year in the fourth and fifth years of the surgery. A repeat MRI was conducted in March 2024, which revealed no significant abnormalities (**Figures 4A1–A4**). Currently, at 24 months after the surgery, denosumab has been used a total of 7 times, the patient has not experienced any significant discomfort, and the follow-up is still ongoing.

## 3 Discussion

GCTB consists mainly of neoplastic stromal cells, multinucleated giant cells, and their monocytic precursors (7). Stromal cells express RANKL, a tumor component of GCTB that stimulates the proliferation of multinucleated giant cells and their monocytic precursor aggregates. Multinucleated giant cells express RANK, which binds to RANKL, disrupting normal bone homeostasis and causing excessive bone resorption (8, 9). Denosumab inhibits osteoclast activation and promotes bone deposition, and its specificity and affinity for RANKL is higher than that of RANK, blocking the RANK/RANKL signaling pathway by binding to RANKL and thus inhibiting the interaction between neoplastic stromal cells and multinucleated giant cells, which is closely associated with tumor recurrence (10).

The valuable role of denosumab in GCTB has gained a consensus, albeit the duration of use for the pharmacological treatment and the timing of its use that can maximize the benefits to the patients remain debatable. The preoperative use of denosumab can make the lesion site more amenable to surgical treatment, especially in patients with larger, destructive, recurrent GCTB (11). Rutkowski et al. (12) reported that the preoperative use of denosumab was effective in reducing the staging of GCTB, with more than one-third (38%) of the patients showing reduced grading of their surgery, which subsequently reduced the need for a more invasive procedure as well as stabilized or improved the neurological status without increasing the risk of neurological deterioration (13). Preoperative adjuvant therapy with denosumab also reduced the blood supply to the tumor tissues and hardened the lesion, thereby facilitating tumor resection (14). In addition, it was effective in improving patients’ pain symptoms, overall functioning, tumor size, and histological features, thereby providing a reference for timing and the certainty of surgery (15).

However, in contrast, a study by Guo et al. (16) found that 43% of the patients who used denosumab preoperatively experienced recurrence, which suggests that the preoperative use of denosumab may increase the risk of local recurrence of GCTB treated with scraping. Moreover, the local recurrence rate after a curettage procedure following the preoperative use of denosumab was significantly higher when compared to that directly after a curettage procedure (17). This difference can be attributed to the fact that although the use of denosumab significantly reduced the tumor size, it increased the risk of recurrence due to the central sclerosis sign and peripheral bone formation. This occurred because of the inadequate scraping of the tumor considering that that the tumor area could not be correctly identified intraoperatively, which ultimately leads to the retention of the tumor tissues (18). GCTB is an aggressive tumor and there is no evidence of blood or lymphatic transmission.

Boriani et al. (19) proposed that denosumab should be used preoperatively for ≥6 months in patients undergoing intra-lesional curettage of the tumor lesion, which can be extended by up to 12 months in patients with an insignificant local response to the tumor. Most authors recommend an estimated duration of 3–6 months, with an initial loading consisting of 120 mg of denosumab administered subcutaneously thrice a week at the start and then continued thereafter at the same rate once a month. The prolonged preoperative use (>9 months) of denosumab has been associated with more surgical complications and a higher risk of vertebral fracture; the prolonged use results in excessive ossification of the original tumor, making it impossible to determine the safe boundaries of tumor resection, which, in turn, increases the risk of local recurrence (20). Currently, short courses of preoperative denosumab have demonstrated effectiveness in reducing surgical grading and in achieving tumor control. Zhang et al. (21) suggested a recurrence rate of 27% in patients undergoing surgery with a short-term (6 doses) denosumab course, stating more potential benefit from shortening the number of preoperative medications when used again. In sacral GCTB, a short course (≤3 doses) of preoperative denosumab treatment not only achieves the local control of the tumor but also improves the likelihood of intraoperative neurological and postoperative functional preservation (22). Relevant imaging evaluations revealed that short-term (≤3 weeks) preoperative neoadjuvant treatment of spinal GCTB with denosumab induces a radiological and histological response that sclerosis the tumor, reduces the soft-tissue component of the tumor, and prevents adhesion to the dura mater, nerve roots, and other important tissue structures, thereby facilitating the achievement of optimal oncological and functional outcomes (23). Ultra-short-term (<3 months) denosumab treatment before the surgery can help achieve the therapeutic effect of a conventional treatment course as well as reduce the risk of local recurrence (14). In a single-center retrospective study by Hindiskere et al. (24), no difference was noted between short (≤3) and long (>3) preoperative courses of denosumab treatment in terms of the MSTS scores, radiological and histological responses, and recurrence-free survival, showing that that drug toxicity response could be achieved at lesser expense (25). Denosumab is also effective in advanced severe GCTB, and a short course of its treatment can make surgery more effective when conducted after a reasonable assessment of the risk of postoperative recurrence (18).

Denosumab inhibits the binding of RANKL to RANK and the maturation and differentiation of osteoclast precursors to osteoblasts. Histological manifestations of GCTB after denosumab treatment include osteoclast disappearance and bone formation (26). However, it does not affect the mesenchymal cells of the tumor, that is, denosumab does not eliminate the tumor cells, but rather only inhibits their activity (27). Mak et al. (28) reported that the RANKL expression was almost eliminated in patients treated with denosumab, but the tumor mesenchymal cells continued to proliferate, which importantly contributed to tumor recurrence. Therefore, preoperative use of denosumab followed by surgical intervention is necessary and the postoperative use of the drug plays an important role in controlling tumor recurrence.

Intra-lesional curettage surgery should be performed, and denosumab should be administered even after surgery and continued for at least 6–12 months after the surgery to reduce tumor recurrence (20). Guo et al. (29) noted that patients undergoing sacral nerve-sparing sacral osteoblastoma curettage with the preservation of the sacral nerves achieved good recurrence-free survival and the continued use of denosumab for 24 months postoperatively was effective in controlling the early recurrence of the tumor. Pharmacological studies have indicated that denosumab has a half-life of approximately 4 weeks and that the inhibitory effect on osteolysis lasts for at least 3 months (30), albeit the optimal dosing schedule for maintenance therapy has not yet been determined. Jianru Xiao et al. (31) suggested that, for patients with spinal GCTB, denosumab should be continued for 2 years postoperatively (120 mg/4 weeks) that the decision to discontinue the drug should be based on the assessment of the risk of recurrence. Denosumab administered at 4-week intervals prevented bone-related events. Extended dosing intervals may reduce toxicity, drug costs, and the number of clinical visits if the efficacy remains unchanged, albeit there is no clear evidence on the efficacy and safety of extended interval use of denosumab (32). Jiang et al. (33) demonstrated that prolonged intervals of denosumab administration provided a similar tumor control and a significantly lower incidence of adverse bone-related events when compared to the standard dose intervals. A very recent study proposed that denosumab, in combination with the targeted drug Sunitinib, can achieve better outcomes (17). Alternatively, with the adjunctive use of radiotherapy, denosumab can be discontinued early for withdrawal, such that good tumor control can be achieved (34), albeit this aspect warrants more in-depth study before any conclusive inference.

Microwave ablation (MWA) induces high-speed vibration of polar molecules in tumor tissues by applying microwave electromagnetic fields, and these molecules collide with friction, which converts kinetic energy into thermal energy, thereby causing irreversible damage or coagulative necrosis of tumor cells (35, 36). Compared to other thermal ablation technologies, MWA can obtain higher heating efficiency, better tissue conductance, and heat conduction (37). Current studies have shown that it has achieved similar efficacy to surgery in solid organ tumors such as early-stage lung cancer and percutaneous puncture for liver cancer (38). As for bone tumors, it can be used as a preoperative adjuvant therapy to effectively reduce intraoperative blood loss and minimize the risk of tumor contamination in the surrounding tissues (39). It effectively manages GCTB with soft tissue extensions, which can be effectively inactivated without damaging the surrounding normal tissues when detected by a pycnometer needle (40). For GCTB occurring in the extremities, using MWA combined with cement-filled internal fixation can maximize the preservation of joint function without violating the articular surface, and the recurrence rate is not significantly different from that of total resection (41). It can maintain the integrity of the joint and achieve biological repair of the bone defect lesion (42). MWA has been used in the treatment of bone tumors for more than 30 years. It can be used as an independent percutaneous minimally invasive treatment for some benign bone tumors and bone metastases, or as an auxiliary treatment for hemostasis, tumor inactivation, or improving the safety of tumor resection boundary (43).

Our analysis revealed that the prolonged use of denosumab preoperatively, the use of a simple curettage without any adjuvant therapy, the lack of continued use of denosumab, or premature discontinuation of denosumab postoperatively were the main contributors to the high recurrence rate of GCTB. The use of adjuvants has now been shown to significantly reduce the recurrence rate of GCTB. Local adjuvants, including high-speed burring, ethanol, and cryosurgery were used in conjunction with intra-lesional scraping to improve the local control aspect. However, due to the special anatomical location of the spine, the use of these adjuvants can greatly increase the risk of spinal cord and nerve damage (44). However, microwave ablation-assisted lesion scraping, by adjusting the working power and controlling the microwave ablation temperature by real-time intraoperative monitoring, can protect the spinal cord, nerves, and other important tissues, as well as maximize the killing and removal of the tumor cells, which has already demonstrated its absolute advantages in the treatment of extremity GCTB (45), while avoiding the risks of other adjuvant therapies.

In summary, in the present case, denosumab was used thrice preoperatively to reduce the size of the tumor and cause local ossification. Intraoperative tumor scraping when assisted by microwave ablation achieved maximum tumor debulking. Tumor control was achieved with the use of denosumab at progressively longer intervals in the postoperative period to taper off the drug, which significantly reduced the rate of tumor recurrence. Thus, the present review and case experience provide a new perspective on the clinical application of denosumab.

## Data Availability

The original contributions presented in the study are included in the article/**Supplementary Material**. Further inquiries can be directed to the corresponding author.
