# Supplementary figures and images for: Denosumab combined with microwave ablation excisional scraping for giant cell tumor of the thoracic spine: a case report and literature review
Source: Front Oncol. 2024 Sep 19;14:1402550. doi: 10.3389/fonc.2024.1402550 (PMC11446914; doi:10.3389/fonc.2024.1402550)

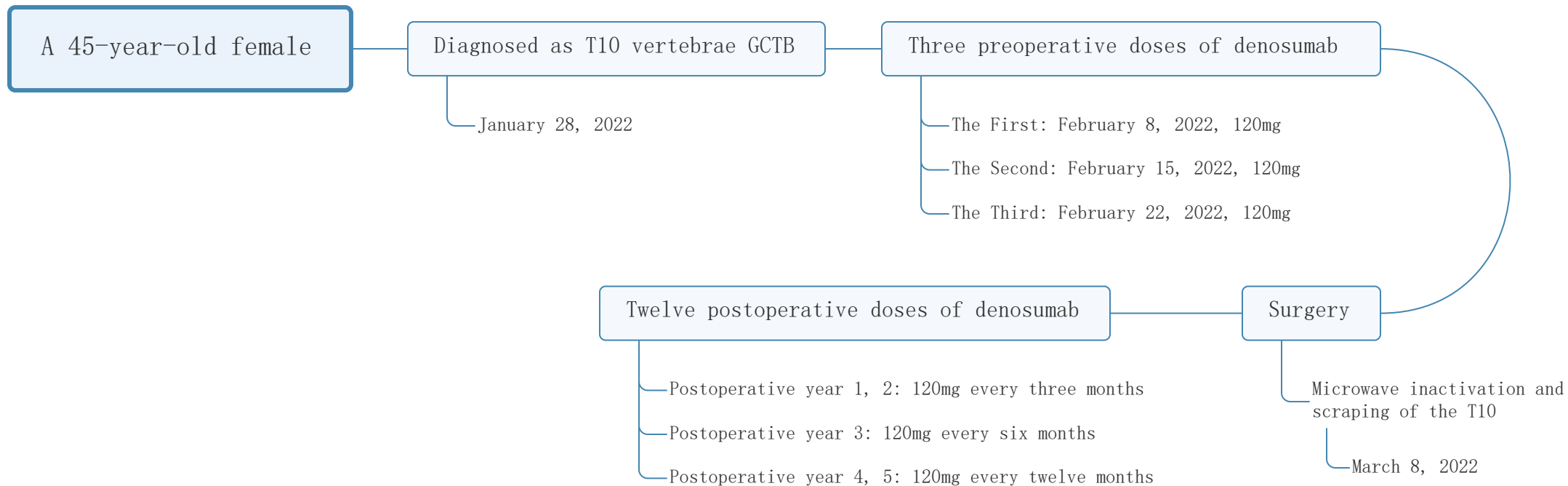

Supplement: Supplementary file 1 [file DataSheet1.pdf]
